# ﻿New species in the *Elachista praelineata* species group (Lepidoptera, Elachistidae, Elachistinae) from East Africa, with identification keys to the Afrotropical species

**DOI:** 10.3897/zookeys.1267.177018

**Published:** 2026-01-20

**Authors:** Virginijus Sruoga, Lauri Kaila

**Affiliations:** 1 Institute of Biosciences, Life Sciences Center, Vilnius University, Saulėtekio Ave. 7, LT-10257 Vilnius, Lithuania Vilnius University Vilnius Lithuania; 2 Zoology Unit, Finnish Museum of Natural History, P.O. Box 17, FI-00014 University of Helsinki, Helsinki, Finland University of Helsinki Helsinki Finland

**Keywords:** Checklist, Ethiopia, identification key, Kenya, Microlepidoptera, mining moths, morphology, Tanzania, taxonomy

## Abstract

Four new species, *Elachista silfverbergi***sp. nov.** from Ethiopia and Kenya, *E. griseifrons***sp. nov.** from Ethiopia, *E. levis***sp. nov.** from Tanzania, and *E. conica***sp. nov.** from Ethiopia are described. The habitus and genitalia are diagnosed and illustrated in detail. Identification keys to the Afrotropical species of the *E. praelineata* species group, based on male and female genitalia, are provided.

## ﻿Introduction

*Elachista* Treitschke, 1833, is a genus of small, often dull-coloured moths, but sometimes showing shiny, metallic markings on forewings. Their larvae are leaf miners on monocotyledons, most commonly grasses and sedges (Parenti and Varalda 1994). It is the most diverse genus within the subfamily Elachistinae and is currently divided into five subgenera and 19 species groups (Kaila 2019). Prior to the addition of the new taxa described herein, the genus comprised 698 recognized species (Kaila et al. 2025; Kaila and Tokár 2026).

The *E. praelineata* species group was delineated by Kaila (1999b, 2000), based on *Elachista praelineata* Braun, 1915, which is known from the Nearctic region. It comprises predominantly dark-coloured moths often with pale transverse fascia near the middle of the forewing and a pair of pale distal spots. In some species, these markings are shiny, metallic, and silvery. Diagnostic features of the male genitalia include an elongate cucullus, a subtle hump along the distal fold of the costa, and a more or less reduced digitate process. Female abdominal tergite VII exhibits a distinctive morphological trait: a prominent tuft of elongated, hair-like scales arising from tergite VII (Kaila 1999b; Sugisima 2005; Sruoga and Havelka 2023). The life history of the species is poorly known. Food plants, all belonging to Poaceae or Cyperaceae, have been recorded for fewer than half of the known species, and only that of *E. trifasciata* (Wollaston, 1879), *Carex dianae* Steud., is documented from the Afrotropical region, St. Helena (Fowler and Karisch 2020).

The Afrotropical species of the *E. praelineata* species group remain poorly explored. Prior to this publication, only four out of the 37 species known in the group had been recorded from the Afrotropical region (De Prins and De Prins 2011–2025). In the present study, four new species are described, and keys to the Afrotropical species are provided.

## ﻿Material and methods

This paper is based on material mainly obtained from the Finnish Museum of Natural History, Helsinki, Finland (**MZH**); one specimen from the Zoological Museum, University of Copenhagen, Denmark (**ZMUC**); and one specimen from the research collection of David J. L. Agassiz, Weston-super-Mare, United Kingdom (**DJLA**).

Adult specimens were examined externally using a Nikon SMZ445 stereomicroscope. The forewing length was measured along the costa from the wing base to the apex of the terminal fringe scales with an ocular micrometer. The head width was measured between the inner edges of the antennal bases. The genitalia were prepared following the standard method described by Robinson (1976) and Traugott-Olsen and Nielsen (1977). The male genital capsule was stained with fuchsin, the abdominal pelt, and the female genitalia with chlorazol black (Direct Black 38/Azo Black). The genital morphology was examined using a Leica DM6 B microscope. The photographs of adults were taken using a Canon EOS 80D camera fitted with a Canon MP-E 65 mm macro lens, attached to a macro rail (MJKZZ Qool Rail). Genitalia photographs were taken with a Leica DM6 B microscope and a Leica K3C digital camera. Zerene Stacker 1.0, with a retouch function, was used for image stacking. All images were optimized and grouped into plates using Adobe Photoshop CC 2019.

The descriptive terminology of morphological structures follows Traugott-Olsen and Nielsen (1977), with some modifications by Kaila (1997, 1999a, 2011). A comparison of the length of the phallus in relation to the valva was measured as the longest line from the base of the sacculus to the apex of the cucullus.

## ﻿Results

### ﻿Checklist of the Afrotropical species of the *Elachista praelineata* species group:

**1. *Elachista conica* sp. nov.**

**Distribution.** Ethiopia.

**Holotype** ♂ in MZH.

**2. *Elachista griseifrons* sp. nov.**

**Distribution.** Ethiopia.

**Holotype** ♂ in MZH.

**3. *Elachista kakamegensis* Sruoga & De Prins, 2009**

*Elachista kakamegensis*Sruoga and De Prins 2009: 8.

**Distribution.** Kenya.

**Holotype** ♂ and 2♀ paratypes in the Royal Museum for Central Africa, Tervuren, Belgium.

**4. *Elachista levis* sp. nov.**

Distribution. Tanzania.

**Holotype** ♂ in ZMUC.

**5. *Elachista merimnaea* Meyrick, 1920**

*Elachista merimnaea* Meyrick, 1920: 297; Parenti 1988: 189.

**Distribution.** South Africa.

**Holotype** ♂ in the South Africa Museum, Cape Town, Republic of South Africa.

**6. *Elachista semophanta* Meyrick, 1914**

*Elachista semophanta* Meyrick, 1914: 281; Sruoga 2002: 138.

**Distribution.** Malawi.

**Holotype** ♂ in the Natural History Museum, London, United Kingdom.

**7. *Elachista silfverbergi* sp. nov.**

**Distribution.** Ethiopia, Kenya.

**Holotype** ♂ and 40 ♂ paratypes in MZH; 1 ♂ paratype in DJLA.

**8. *Elachista trifasciata* (Wollaston, 1879)**

*Stagmatophora trifasciata* Wollaston, 1879: 437.

*Elachista trifasciata* (Wollaston); Sinev 2002: 164; Fowler and Karisch 2020: 28

**Distribution.** Saint-Helena.

**Syntypes** (12) in the Natural History Museum, London, United Kingdom.

### ﻿Key to the Afrotropical species of the *Elachista praelineata* species group

Based on male genitalia (male of *E. semophanta* is unknown).

**Table:** 

1	Uncus lobes are reduced, very small, like a pustule (Fowler and Karisch 2020, fig. 12)	***E. trifasciata***
–	Uncus lobes well developed, large, widely apart from each other	**2**
2	Vesica with two longitudinal, weakly sclerotized bands (Sruoga and De Prins 2009, figs 18, 19)	***E. kakamegensis***
–	Vesica without any sclerotized formations	**3**
3	Phallus with a band of proximally directed small spines in apical 1/3	**4**
–	Phallus without spines in apical 1/3	**6**
4	Digitate process short and triangularly shaped (Fig. 3A, D)	***E. griseifrons***
–	Digitate process narrow and long, at least 5 times as long as its width	**5**
5	Spinose knob of gnathos large, rounded (Fig. 2A)	***E. silfverbergi***
–	Spinose knob of gnathos reduced, not rounded (Fig. 4A, D)	***E. conica***
6	Digitate process short and triangularly shaped; phallus with hooked apex (Fig. 3E–G)	***E. levis***
–	Digitate process narrow and long; apex of phallus acute, but not hooked (Parenti 1988, figs 12–15)	***E. merimnaea***

### ﻿Key to the Afrotropical species of the *Elachista praelineata* species group

Based on female genitalia (females of *E. conica* sp. nov., *E. griseifrons* sp. nov., *E. levis* sp. nov., *E. merimnaea*, and *E. silfverbergi* sp. nov. are unknown).

**Table:** 

1	Corpus bursae with signa (Sruoga 2002, figs 3, 4)	***E. semophanta***
–	Corpus bursae without signa	**2**
2	Antrum short, bowl-shaped, membranous, without any sclerotized formations; colliculum narrow, strongly sclerotized (Fowler and Karisch 2020, fig. 13)	***E. trifasciata***
–	Antrum long, gradually tapering anteriorly, with two prolonged sclerotized bands; colliculum not pronounced (Sruoga and De Prins 2009, figs 22–24)	***E. kakamegensis***

#### ﻿ Elachista silfverbergi sp. nov.

Taxon classificationAnimaliaLepidopteraElachistidae

520C9D2C-F054-5777-A5C8-2DE0433D796D

https://zoobank.org/E0A98868-9BAB-4412-9B4B-C8E42B63811C

Figs 1A, B, 2, 4E

##### Material examined.

***Holotype*.** Ethiopia • ♂; Addis Ababa; 8.9506°N, 38.8256°E; 3–4 Feb. 1974; H. Silfverberg leg.; gen. prep. VS622; MZH. ***Paratypes*.** Ethiopia • 7 ♂; Addis Ababa; 8.9506°N, 38.8256°E; 30 Jan.–1 Feb. 1974; H. Silfverberg leg.; gen. prep. L. Kaila 1897, 1898, 1899, VS617; MZH • 2 ♂; same data except date; 1–3 Feb. 1974; gen. prep. L. Kaila 1900, VS641 • 1 ♂; same data except date; 3–4 Feb. 1974; gen. prep. VS621 • 1 ♂; same data except date; 4–5 Feb. 1974; gen. prep. VS640 • 8 ♂; same data except date; 21–23 Feb. 1974; gen. prep. VS624, VS625, VS629 • 4 ♂; same data except date; 23–25 Feb. 1974; gen. prep. VS626, VS631 • 3 ♂; same data except date; 25–27 Feb. 1974; gen. prep. VS620, VS642 • 12 ♂; same data except date; 18–19 Mar. 1974; gen. prep. L. Kaila 1896, VS618, VS619, VS628, VS630 • 1 ♂; same data except date; 20–21 Mar. 1974; gen. prep. VS623 • 1 ♂; Arba Minch; 6.03°N, 37.54°E; 28 Feb.–1 Mar. 1974; gen. prep. L. Kaila 1387; Mzh. Kenya • 1 ♂; Rift Valley, Prov. Turi; 8000 ft; 4. Mar 1999; DJL Agassiz leg.; gen. prep. L. Kaila 850; DJLA.

##### Diagnosis.

Among the known Afrotropical species of the *E. praelineata* species group, *E. silfverbergi* can be compared with *E. merimnaea* Meyrick, 1920, known from South Africa (for external characters and male genitalia, see Parenti 1988, figs 4, 12–15). However, in contrast to *E. silfverbergi*, moths of *E. merimnaea* are lightly coloured, the cucullus of the valva is blunt, the phallus lacks a band of spines, and the apex of the phallus is not hooked as in *E. silfverbergi*. *Elachista silfverbergi* also resembles *E. amamii* Parenti, 1983, known from Japan and Thailand (for external characters and male genitalia, see Parenti 1983, table 2, Sugisima 2005, figs 1, 2, 15, 39), and *E. simulans* Sruoga, 2022, known from Nepal (for external characters and male genitalia, see Sruoga 2022, figs 24–27). However, unlike *E. silfverbergi*, in those species the saccus is prominent and narrow, the phallus lacks a band of spines, and the apex of the phallus is not as hooked as in *E. silfverbergi*.

##### Description.

**Figure 1.**
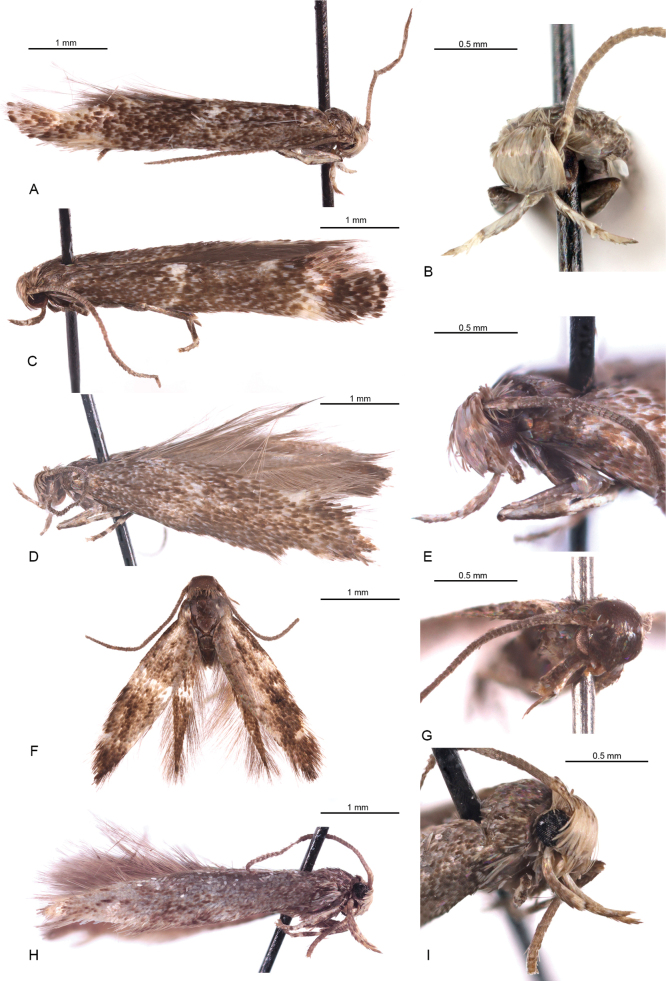
*Elachista* species. **A***E. silfverbergi* sp. nov., adult male, holotype; **B** ibid., head, frontal view; **C***E. silfverbergi* sp. nov., adult male, paratype; **D***E. griseifrons* sp. nov., adult male, holotype; **E** ibid., head, lateral view; **F***E. levis* sp. nov., adult male, holotype; **G** ibid., head, fronto-lateral view; **H***E. conica* sp. nov., adult male, holotype; **I** ibid., head, fronto-lateral view.

**Male.** (Fig. 1A, B). Forewing length 3.6–4.5 mm; wingspan 7.8–9.4 mm (*N = 42*). ***Head***: frons white to off-white, vertex whitish-grey intermixed with brownish-grey, some scales with darkened tips, neck tuft brownish-grey; labial palpus 1.3–1.5 times as long as width of head, off-white above and greyish-brown below; scape and first flagellomere blackish-brown, flagellum brownish-grey, pecten off-white. ***Thorax***, tegula, and forewing strongly mottled with scales, basally off-white and distally grey-brown; antemedian transverse fascia white, blurred, interrupted at middle, present as two small white spots near costal and dorsal margins; small costal and tornal spots blurred, off-white; fringe brownish-grey. Hindwing and its fringe brownish-grey.

**Female.** Unknown.

**Figure 2.**
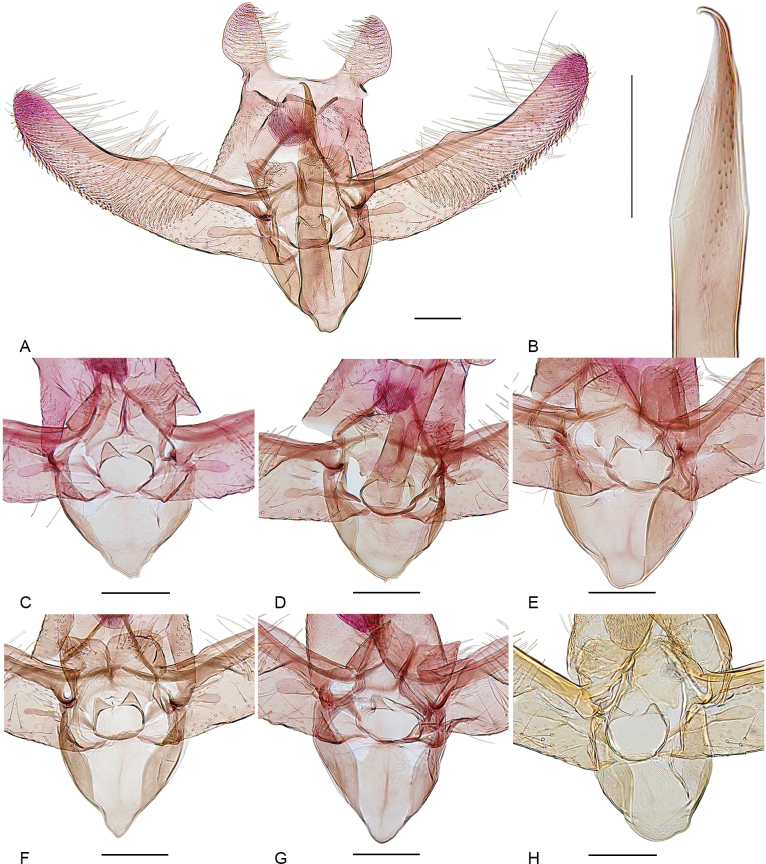
Male genitalia of *Elachista silfverbergi* sp. nov. **A** general view, holotype, gen. prep. VS622; **B** distal part of phallus, paratype, gen. prep. 624; **C–H** juxta region: **C** paratype, gen. prep. VS620; **D** paratype, gen. prep. VS621; **E** paratype, gen. prep. VS625; **F** paratype, gen. prep. VS626; **G** paratype, gen. prep. VS641; **H** paratype, gen. prep. 850. Scale bars: 0.1 mm.

**Male genitalia** (Fig. 2). Uncus lobes widely apart from each other, longer than wide, broadest before middle, tapered distally, setae on ventral surface dense, scale-like near apex and along lateral margin, becoming longer and thinner in mediobasal part. Socius with a few long setae. Spinose knob of gnathos rounded, about as large as width of uncus lobe at its widest point. Valva long and narrow, about four times as long as wide at costal hump; basal fold of costa extended to 1/2 of valva, beyond it with small, broad hump; cucullus long, slightly bent towards costa. Median plate of juxta with a pair of dorsally directed lateral pockets. Juxta lobes large, median margin straight, joining straight distal margin at right angle, distal margin convex, ventral surface distally with long setae. Digitate process wide basally, nearly parallel or slightly varying in shape from middle, sometimes with single seta near tip (Fig. 2C). Vinculum V-shaped, saccus short, not prominent, varies slightly in length and width, with weak median ridge in some specimens. Phallus about 0.7 as long as valva, weakly bent before middle, caecum small, apex acute, and curved to form strongly sclerotized hook; ventral surface with band of proximally directed small spines in apical 1/3; vesica without cornuti.

##### Biology.

Unknown.

##### Flight period.

Adults were collected from early January until early March.

##### Distribution.

Central and south-western Ethiopia and south-western Kenya.

##### Etymology.

The species is named after the Finnish entomologist Hans Silfverberg, who collected most type material.

#### ﻿ Elachista griseifrons sp. nov.

Taxon classificationAnimaliaLepidopteraElachistidae

1D0B3564-719D-5B4F-B16E-43654072EF0E

https://zoobank.org/3F70F486-E38B-455D-B5E3-79F7453D0421

**Figure 3.**
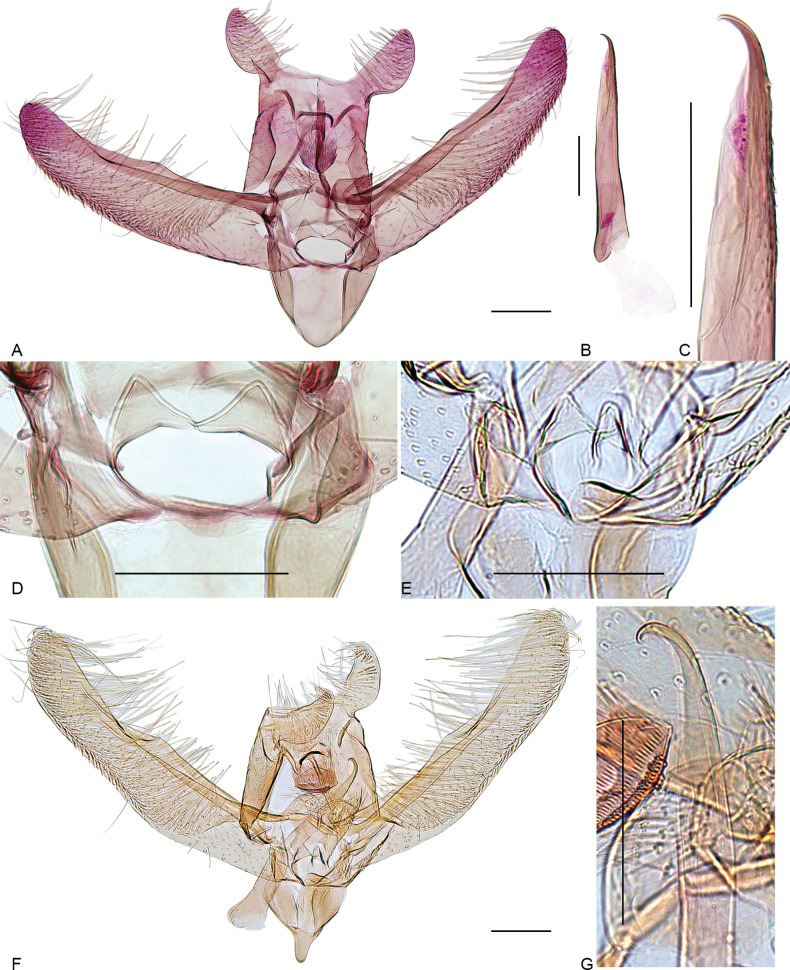
Male genitalia of *Elachista* species. **A–D***E. griseifrons* sp. nov., holotype: **A** general view, phallus removed, gen. prep. VS627; **B** phallus, gen. prep. VS627; **C** ibid., distal part; **D** juxta region, gen. prep. VS627; **E–G***E. levis* sp. nov., holotype, gen. prep. 1514: **E** juxta region; **F** general view; **G** distal part of phallus. Scale bars: 0.1 mm.

**Figure 4.**
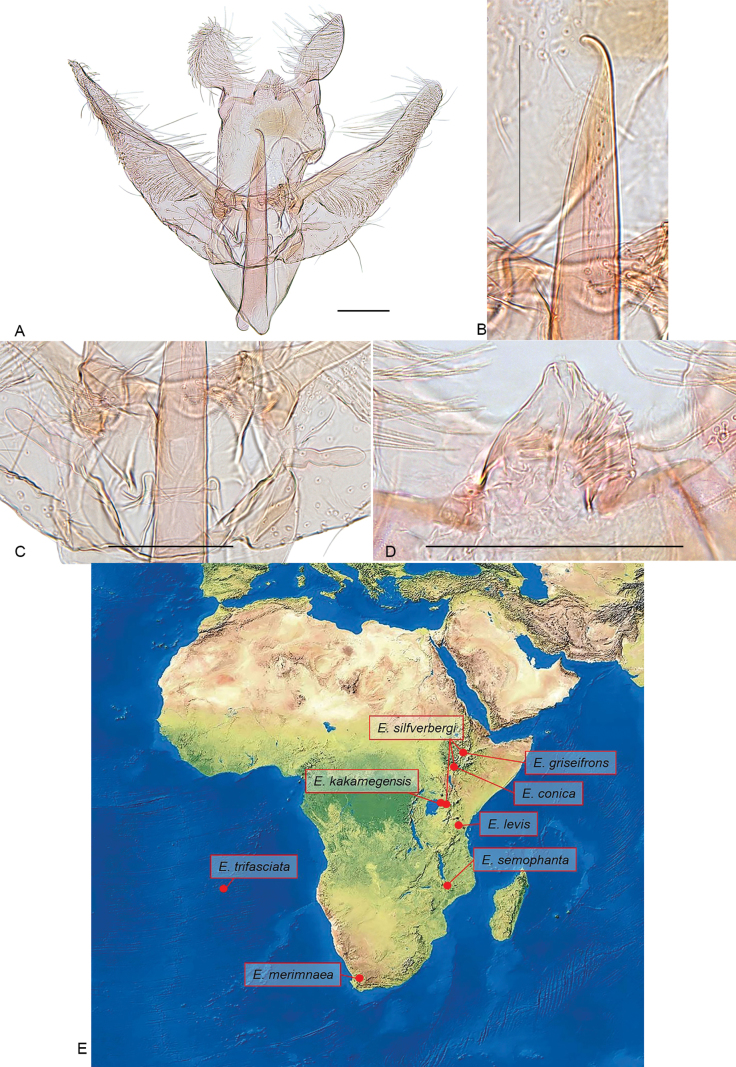
Male genitalia and distribution map of *Elachista* species. **A–D** male genitalia of *E. conica* sp. nov., holotype, gen. prep. 1388: **A** general view; **B** distal part of phallus; **C** juxta region; **D** gnathos; **E** Afrotropical distribution of *E. praelineata* species group. The map was made using Google Maps. Scale bars: 0.1 mm.

Figs 1D, E, 3A–D, 4E

##### Material examined.

***Holotype*.** Ethiopia • ♂; Addis Ababa; 8.9506°N, 38.8256°E; 1–3 Feb. 1974; H. Silfverberg leg.; gen. prep. VS627; MZH.

##### Diagnosis.

*Elachista griseifrons* externally closely resembles *E. silfverbergi*, known from the same locality, but it differs in a more grey frons and longer labial palpus compared to the width of the head. Also, the male genitalia are very similar to those of *E. silfverbergi*. However, the digitate process is reduced to a bluntly triangular lobe in *E. griseifrons*, whereas in *E. silfverbergi* it is well developed and about twice as long as in *E. griseifrons*.

##### Description.

**Male.** (Fig. 1D, E). Forewing length 3.9 mm; wingspan 8.4 mm (*N = 1*). ***Head***: frons whitish-grey, vertex greyish-brown intermixed with whitish-grey, some scales with darkened tips, neck tuft greyish-brown; labial palpus 1.75 times as long as width of head, whitish-grey above and greyish-brown below; scape, pecten, and flagellum brownish-grey. ***Thorax*** dark grey-brown, tegula grey-brown. Forewing: strongly mottled with scales basally off-white and distally grey-brown; wing slightly darker beyond middle; antemedian transverse fascia blurred, incomplete, present as small, greyish-white spot at fold, small costal and tornal spots blurred, greyish-white; fringe brownish-grey. Hindwing and its fringe brownish-grey.

**Female.** Unknown.

**Male genitalia** (Fig. 3A–D). Uncus lobes widely apart from each other, longer than wide, broadest before middle, tapered distally, setae on ventral surface dense, scale-like near apex and along lateral margin, becoming longer and thinner in mediobasal part. Socius with a few long setae. Spinose knob of gnathos large and rounded, as large as width of uncus lobe at its widest point, proximal margin shortly pointed. Valva long and narrow, about four times as long as wide at costal hump; basal fold of costa extended to 1/2 of valva, beyond it with small, broad hump; cucullus long, slightly bent towards costa. Median plate of juxta with a pair of large dorsally directed lateral pockets. Juxta lobes large, separated from each other with shallow incision; median margin straight, joining straight distal margin at right angle, distal margin convex, ventral surface distally with long setae. Digitate process glabrous, reduced to a small, bluntly triangular lobe. Vinculum V-shaped, saccus not prominent. Phallus about 0.7 as long as valva, straight, caecum small, apex acute and curved to form strongly sclerotized hook; ventral surface with band of proximally directed small spines in apical 1/3; vesica without cornuti.

##### Biology.

Unknown.

##### Flight period.

The single specimen was collected in early February.

##### Distribution.

So far, this species is known only from central Ethiopia.

##### Etymology.

From the Latin *griseus*, meaning “grey” and *frons*, meaning “forehead”, in reference to the grey forehead of the moth. The name is a noun in apposition.

##### Remarks.

The forewings of this specimen are somewhat abraded. Therefore, the description is approximate.

#### ﻿ Elachista levis sp. nov.

Taxon classificationAnimaliaLepidopteraElachistidae

ED9310F7-7A59-5F01-A654-E7A31754198B

https://zoobank.org/98956FCA-3E8B-4D4F-B2B9-0586F092522E

Figs 1F, G, 3E–G, 4E

##### Material examined.

***Holotype*.** Tanzania • ♂; West Usambara Mts, Mazumbai; 1600 m; 01 Aug. 1980; M. Stoltze & N. Scharff leg.; gen. prep. L. Kaila 1514; ZMUC.

##### Diagnosis.

The male genitalia of *E. levis* closely resemble those of *E. silfverbergi*, known from Ethiopia and Kenya, and *E. griseifrons*, known from Ethiopia. However, *E. levis* lacks a band of spines in the apical part of the phallus and has a vinculum with a long, narrow saccus. Additionally, *E. levis* differs from *E. silfverbergi* by having a short, triangular digitate process.

##### Description.

**Male.** (Fig. 1F, G). Forewing length 2.6 mm; wingspan 5.7 mm (*N = 1*). ***Head***: labial palpus 1.25 times as long as width of head, whitish-grey above and greyish-brown below; scape and pecten brownish-grey, flagellum brownish-grey, with very short cilia. ***Thorax*** dark grey-brown, tegula brownish-grey. Forewing: mottled with scales basally whitish-grey and distally grey-brown; wing slightly darker beyond middle; antemedian transverse fascia blurred, small costal and tornal spots blurred, greyish-white; fringe grey-brown. Hindwing and its fringe greyish-brown.

**Female.** Unknown.

**Male genitalia.** (Fig. 3E–G). Uncus lobes widely apart from each other, broadest in basal part, longer than wide, tapered distally, setae on ventral surface dense, scale-like near apex and along lateral margin, becoming longer and thinner in mediobasal part. Socius with a few short setae. Spinose knob of gnathos large and oval, slightly larger than width of uncus lobe at its widest point. Valva long and narrow, about five times as long as wide at costal hump; basal fold of costa extended to 1/2 of valva, beyond it with small, broad hump; cucullus long, slightly bent towards costa. Median plate of juxta with a pair of large dorsally directed lateral pockets. Juxta lobes large, median margin straight, joining straight distal margin at right angle, distal margin convex, ventral surface distally with long setae. Digitate process glabrous, reduced to a small, bluntly triangular lobe. Vinculum V-shaped, saccus prominent, long, and narrow. Phallus about 0.6 as long as valva, weakly bent before middle, caecum small, apex acute, and curved to form strongly sclerotized hook; vesica without cornuti.

##### Biology.

Unknown.

##### Flight period.

The single specimen was collected in early August.

##### Distribution.

So far, this species is known only from north-eastern Tanzania.

##### Etymology.

From the Latin *levis*, meaning “smooth, polished” in reference to the phallus without coarse spines on the surface.

##### Remarks.

The holotype moth is somewhat abraded. Therefore, the description is approximate.

#### ﻿ Elachista conica sp. nov.

Taxon classificationAnimaliaLepidopteraElachistidae

064C00F3-5263-5518-A449-6170E0BCF41D

https://zoobank.org/A8652E69-4FB5-4302-9E9F-DF031D560D1B

Figs 1H, I, 4

##### Material examined.

***Holotype*.** Ethiopia • ♂; Gemu Gofa, Arba Minch; 6.03°N, 37.54°E; 28 Feb.–1 Mar. 1974; H. Silfverberg leg.; gen. prep. L. Kaila 1388; MZH.

##### Diagnosis.

Among the species of the *E. praelineata* species group, *E. conica* can be readily separated by its peculiar shape of the valva with a narrow, tapered cucullus and a strongly reduced spinose knob of the gnathos. As such, *E. conica* cannot be confused with any other known species of the *E. praelineata* species group.

##### Description.

**Male.** (Fig. 1H, I). Forewing length 4.0 mm; wingspan 8.6 mm (*N = 1*). ***Head***: frons yellowish-white, vertex, and neck tuft yellowish-white, with few brownish-grey tipped scales; labial palpus 1.4 times as long as width of head, yellowish-white above and brownish-grey below; antenna brownish-grey, in distal half with slightly raised scales and weakly annulated with paler rings. ***Thorax***, tegula, and forewing ground colour greyish-white, variably covered by greyish-brown tipped scales; fringe brownish-grey. Hindwing and its fringe brownish-grey.

**Female.** Unknown.

**Male genitalia.** (Fig. 4A–D). Uncus lobes widely apart from each other, broadest in basal part, longer than wide, tapered distally, setae on ventral surface dense, scale-like near apex and along lateral margin, becoming longer and thinner in mediobasal part. Socius with a few long setae. Spinose knob of gnathos very small and weakly sclerotized, in shape of two drop-like lobes. Valva broadest in basal part, about four times as long as wide at costal hump; basal fold of costa extended to 1/2 of valva, beyond it with small, inconspicuous hump; cucullus long and narrow, gradually tapered towards apex. Median plate of juxta with a pair of large dorsally directed lateral pockets. Juxta lobes large, separated from each other with shallow incision; median margin straight, joining straight distal margin at right angle, distal margin convex, ventral surface distally with long setae. Digitate process glabrous, almost parallel-sided. Vinculum V-shaped, saccus short, not prominent. Phallus about 0.8 as long as valva, straight, caecum narrow and long, apex acute, and curved to form strongly sclerotized hook; ventral surface with band of proximally directed small spines in apical 1/3; vesica without cornuti.

##### Biology.

Unknown.

##### Flight period.

The single specimen was collected at the end of February or in early March.

##### Distribution.

So far, this species is known only from south-western Ethiopia.

##### Etymology.

From the Latin *conica*, the feminine form of the adjective *conicus*, meaning “conical” in reference to the shape of the valva.

##### Remarks.

The holotype moth is somewhat abraded. Therefore, the description is approximate.

## ﻿Discussion

Our current research is based on the morphology of the adult males. Attempts to extract DNA from the studied material were unsuccessful, likely due to the considerable time elapsed since the specimens were collected.

Including the species described herein, the *Elachista praelineata* species group now comprises 41 species, distributed across all continents except Antarctica, with the highest species diversity reported in the Palearctic and Nearctic regions (Sruoga and Havelka 2023). In the Afrotropics, the number of described species within this group has doubled—from four to eight—with most species occurring in the eastern and southern regions (Fig. 4E). However, these findings are likely due to uneven geographic sampling rather than representing a true pattern of species diversity. As large areas of tropical Africa remain underexplored, the actual number of species in the *E. praelineata* species group is likely to be considerably higher.

## Supplementary Material

XML Treatment for Elachista silfverbergi

XML Treatment for Elachista griseifrons

XML Treatment for Elachista levis

XML Treatment for Elachista conica

## ﻿Additional information

### Use of AI

No use of AI was reported.

### Author ORCIDs

Virginijus Sruoga https://orcid.org/0000-0002-5687-8299

Lauri Kaila https://orcid.org/0000-0003-0277-1872

### Data availability

All of the data that support the findings of this study are available in the main text.

## References

[B1] De Prins J De Prins W (2011–2025) Afromoths, online database of Afrotropical moth species (Lepidoptera). World Wide Web electronic publication. http://www.afromoths.net [accession date 04-11-2025]

[B2] Fowler L Karisch T (2020) *Elachista trifasciata* (Wollaston, 1879) on St Helena Island (Lepidoptera, Elachistidae, Elachistinae). Metamorphosis 31(1): 28–32. 10.4314/met.v31i1.7

[B3] Kaila L (1997) A revision of the Nearctic species of *Elachista* s. l. II. The argentella group (Lepidoptera, Elachistidae). Acta Zoologica Fennica 206: 1–93.

[B4] Kaila L (1999a) Phylogeny and classification of the Elachistidae s. s. (Lepidoptera: Gelechioidea). Systematic Entomology 24(2): 139–169. 10.1046/j.1365-3113.1999.00069.x

[B5] Kaila L (1999b) A revision of the Nearctic species of the genus *Elachista**s. l.* III. The bifasciella, *praelineata*, saccharella and freyerella groups (Lepidoptera, Elachistidae). Acta Zoologica Fennica 211: 1–235.

[B6] Kaila L (2000) A review of the South American Elachistidae s. str. (Lepidoptera, Gelechioidea), with descriptions of 15 new species. Steenstrupia (Copenhagen) 25: 159–193.

[B7] Kaila L (2011) Elachistine Moths of Australia (Lepidoptera: Gelechioidea: Elachistidae). Monographs on Australian Lepidoptera (Vol. 11). CSIRO Publishing, Melbourne, 443 pp. 10.1071/9780643103481

[B8] Kaila L (2019) An annotated catalogue of Elachistinae of the World (Lepidoptera: Gelechioidea: Elachistidae. Zootaxa 4632(1): 1–23. 10.11646/zootaxa.4632.1.1 31712495

[B9] Kaila L Tokár Z (2026) *Elachista dimorphella* sp. nov., the first known sexually dimorphic species of *Aphelosetia*, a subgenus of *Elachista* (Elachistidae, Elachistinae). Nota Lepidopterologica 49: 1–15. 10.3897/nl.49.174091

[B10] Kaila L Nupponen K Sruoga V (2025) On species of the *Elachista bedellella* group (Gelechioidea, Elachistidae, Elachistinae) similar with *E. rudectella* Stainton, 1851 and *E. graeca* Parenti, 2002, with descriptions of five new species. Nota Lepidopterologica 48: 103–136. 10.3897/nl.48.142483

[B11] Meyrick E (1914) Exotic Microlepidoptera 1(9). Taylor and Francis, London, 32 pp. [pp. 257–288]

[B12] Meyrick E (1920) Descriptions of South African Micro-Lepidoptera. Annals of the South African Museum 17(4): 273–318. 10.5962/bhl.part.22317

[B13] Parenti U (1983) Elachistidi del Giappone (Lepidoptera, Elachistidae). Bollettino del Museo Regionale di Scienze Naturali – Torino 1(1): 1–20.

[B14] Parenti U (1988) About some African and Asiatic species of the family Elachistidae (Lepidoptera) described by E. Meyrick. Stapfia 16: 185–198.

[B15] Parenti U Varalda PJ (1994) Gli Elachistidi (Lepidoptera, Elachistidae) e le loro piante ospiti. Bollettino del Museo Regionale di Scienze Naturali – Torino 12(1): 73–136.

[B16] Robinson GS (1976) The preparation of slides of Lepidoptera genitalia with special reference to the Microlepidoptera. Entomologist’s Gazette 27: 127–132.

[B17] Sinev SY (2002) World catalogue of Cosmopterigid moths (Lepidoptera: Cosmopterigidae). Trudy Zoologicheskogo Instituta 293: 1–182.

[B18] Sruoga V (2002) Re-descriptions of two Meyrick’s types of *Elachista* (Lepidoptera, Elachistidae) from the Sub-Saharan Africa. Deutsche Entomologische Zeitschrift 49(1): 137–141. 10.1002/mmnd.20020490110

[B19] Sruoga V (2022) New species and records of Elachistinae (Lepidoptera: Gelechioidea, Elachistidae) from Nepal. Zootaxa 5100(4): 573–584. 10.11646/zootaxa.5100.4.7 35391058

[B20] Sruoga V De Prins J (2009) The Elachistinae (Lepidoptera: Elachistidae) of Kenya with descriptions of eight new species. Zootaxa 2172(1): 1–31. 10.11646/zootaxa.2172.1.1

[B21] Sruoga V Havelka J (2023) Review of the Neotropical species of the *Elachista praelineata* species group (Lepidoptera, Elachistidae, Elachistinae) with identification keys and description of a new species from Bolivia. Insects 2023(14): e62. 10.3390/insects14010062 PMC9863831 36661990

[B22] Sugisima K (2005) A revision of the *Elachista praelineata*-group (Lepidoptera, Elachistidae) in Japan, with comments on morphology of the pupa in *Elachista*. Tijdschrift voor Entomologie 148(1): 1–19. 10.1163/22119434-900000160

[B23] Traugott-Olsen E Nielsen ES (1977) The Elachistidae (Lepidoptera) of Fennoscandia and Denmark. Fauna Entomologica Scandinavica 6: 1–299. 10.1163/9789004273290

[B24] Wollaston TV (1879) Notes on the Lepidoptera of St. Helena, with descriptions of new species. Annals & Magazine of Natural History 3(5): 415–441. 10.1080/00222937908562413

